# The Implementation Process of Nurse Prescribing in Poland – A Descriptive Study

**DOI:** 10.3390/ijerph17072417

**Published:** 2020-04-02

**Authors:** Agnieszka Zimmermann, Ewa Cieplikiewicz, Piotr Wąż, Aleksandra Gaworska-Krzemińska, Paweł Olczyk

**Affiliations:** 1 Department of Medical and Pharmaceutical Law, Faculty of Health Sciences with Institute of Maritime and Tropical Medicine, Medical University of Gdańsk, 80-210 Gdańsk, Poland; ewa.cieplikiewicz@gumed.edu.pl; 2 Department of Nuclear Medicine, Faculty of Health Sciences with Institute of Maritime and Tropical Medicine, Medical University of Gdańsk, 80-210 Gdańsk, Poland; piotr.waz@gumed.edu.pl; 3 Department of Nursing Management, Faculty of Health Sciences with Institute of Maritime and Tropical Medicine, Medical University of Gdańsk, 80-210 Gdańsk, Poland; aleksandra.gaworska-krzeminska@gumed.edu.pl; 4 Department of Clinical Chemistry and Laboratory Diagnostics, School of Pharmacy and Division of Laboratory Medicine in Sosnowiec, Medical University of Silesia in Katowice, 41-200 Katowice, Poland; polczyk@sum.edu.pl

**Keywords:** nurse prescribing, nursing practice, nurse supplementary prescribing, legislation

## Abstract

The study aimed to investigate the situation of nurse prescribing, introduced in Poland in 2016, by analyzing the opinions of nurses, expected to be influential on nurses’ actual practices, in response to legislative change to enable nurses to prescribe and comparing this with actual nurse prescribing behaviours during the early years of the legislation. The paper fills a knowledge gap and provides baseline data analysis for subsequent research. Nurses’ opinions were collected during the period they were preparing themselves for prescribing. That data was compared with data on the character and extent of nurses’ actual prescribing practices over the first two years of implementation. The study showed the number of nurse prescriptions increased. Comparing the first and second years of nurse prescribing, the number of nurse independent prescriptions more than doubled. Over the same period, the number of nurse supplementary prescriptions increased almost six-fold. The implementation of nurse prescribing has increased the scope of nursing care, especially in the treatment of the infections, pain and chronic conditions in the elderly.

## 1. Introduction

Traditionally, the task of prescribing medicines has been the domain of physicians but the international trend of growing demands for healthcare services, increasing budget constraints on healthcare delivery and efforts to extend the scope of nursing practices have contributed to demands for nurse prescribing. The practice was first introduced in Idaho, in the United States of America, in 1969 [1]. Since then, nurses have gained the right to prescribe in more than 14 countries, including Australia, Canada, Ireland, Israel, Iran, New Zealand, Norway, Spain, Sweden, the Netherlands, the Republic of South Africa, the United Kingdom, the United States of America and, since 2016, in Poland. When permitted by legislation and qualified, nurse prescribers are responsible for the clinical assessment of patients, for establishing diagnoses and for decisions about the appropriateness of medications, treatments or applications, including the issuing of prescriptions. Usually the practice of nurse prescribing is permitted for a limited list of active substances. This scope of prescribing is either called ‘initial’, ‘independent’ or ‘substitutive’ [2] and in our paper we refer to it as ‘nurse independent prescribing’. Another type of prescribing is practised by ‘supplementary’ prescribers: either nurses or pharmacists; who practise in a voluntary partnership with an independent prescriber, either a doctor or a dentist. Here, the nurse supplementary prescriber collaborates or consults with the independent prescriber before issuing the prescription [3], and in our paper we refer to these as ‘nurse supplementary prescribers’. Nurse prescribing is described in the literature as a necessary piece for consolidating and improving the jigsaw puzzle of the professional nursing identity [4].

New legal provisions for nurse prescribing were introduced in Poland in January 2016. In the absence of any literature on the Polish situation of nurse prescribing, the present study involved an analysis of nurses and midwives opinions immediately prior to the implementation of legal nurse prescribing (2015), and the actual prescribing behaviours and trends of nurses and midwives in the two years immediately following the new law coming into force (2016 and 2017). These two phases formed one integrated whole because, though nurse prescribing was permitted from January 2016, it was not mandatory and prescribing activity was an option at the discretion of suitably qualified nurses and midwives. As such, it was expected that the opinions of nurses may have a direct bearing on their actual prescribing practices; and therefore, the relationship between the two sets of data needed to be understood through an integrated analysis of the before-and-after phasing of the research. Though it is true that in 2016 and 2017, more nurses also finished the nurse prescribing course that we had surveyed in 2015, and that therefore the number of prescribers increased, those from the first phase of the study did become prescribers and the analyzed data also derived from their prescribing. Also, the rationale for our retrospective analysis was because this was the only way could have data to analyze from the whole country for the whole two year period.

In Poland, the new provisions authorized nurses and midwives to prescribe (decide on the choice of medication) and to issue prescriptions for medicines and foods for special dietary purposes, to give referrals for a range of specified diagnostic tests, and to issue orders and prescriptions for reimbursable medical devices. The new entitlement for Polish nurses and midwives takes two forms:

a) Nurse independent prescribing and issuing prescriptions, defined as an element of the independent provision of preventive, diagnostic, therapeutic and rehabilitation services; and
b) Issuing a prescription which is a continuation of medical prescribing (‘nurse supplementary prescribing’), defined as an element of the fulfilment of a doctor’s orders in the process of diagnosis, treatment and rehabilitation.

The entitlement of Polish nurses and midwives to practise as either independent or supplementary prescribers is dependent on their level of tertiary education in nursing or obstetrics, or a specialist’s qualification. Nurses and midwives are permitted to act as independent prescribers and/or supplementary prescribers if, on the one hand, they are master’s graduates (having graduated prior to 2019) who have subsequently completed the relevant specialist ‘Nurse Prescribing Course’ (which was required prior to 2019, but not after), or, alternatively, if they are master’s graduates who graduated after 2019 (and thus did not require additional specialist training because it was integrated into the master’s program). It should be noted that if a nurse or midwife is permitted to prescribe on the basis of either of the two qualification routes stated above, they may, from day to day, either perform as an independent prescriber, or a supplementary prescriber, depending on the practice situation, where sometimes the nurse might act by herself, independently, and at another time, by cooperating with a doctor, be acting as a supplementary prescriber. Nurses and midwives are limited to acting as supplementary prescribers if they are bachelor’s or master’s degree graduates (graduating prior to 2019) who have subsequently completed the relevant specialist ‘Nurse Prescribing Course’, or, if they are bachelor’s degree graduates (graduating since 2019), or, if they are graduates of Poland’s former professional (medical) schools having completed eight years of education plus a further two year specialization (e.g., as a pediatric nurse, surgery nurse) and if they have subsequently completed the relevant specialist ‘Nurse Prescribing Course’. There are two different ‘Nurse Prescribing Courses’ established by the Minister of Health: one for independent prescribing and another for supplementary prescribing. These courses, with practice and theory components, are delivered by medical universities throughout the country and graduates are awarded a Diploma that permits them to prescribe.

Among high-income countries worldwide, Australia, Canada, New Zealand and the US have implemented laws granting the prescribing of a wide range of medicines to advanced practice nurses (APN) with a master’s degree [5]. The extent of prescribing rights in different countries ranges from nearly all medicines within nurses’ specializations (Ireland for nurse prescribers, Netherlands for nurse specialists, UK for nurse independent prescribers) to a limited set of medicines (Cyprus, Denmark, Estonia, Finland, France, Norway, Spain, Sweden) [6]. While nurses in the United Kingdom initially could only prescribe from the Formulary for Nurse Prescribers, currently they may independently prescribe more than 250 different active substances, including controlled drugs [7]. In some countries, for example in Uganda, nurses even have the right to prescribe morphine [8].

In Poland, suitably qualified nurses and midwives (as described above) may, as part of the fulfilment of a doctor’s orders during the patient’s diagnosis, treatment and rehabilitation, issue prescriptions as a continuation of medical prescriptions. In the main, nurse independent prescribers in Poland may prescribe specific medical devices, medicinal food and medicinal products with specific active substances, except for medicines containing very potent substances or controlled drugs. The above mentioned ‘specific’ medications should be understood as referring exclusively to those medicines, devices and products listed in the regulations of the Minister of Health. The regulations list 31 active substances, including: anti-emetics, anti-infective medications for topical use, gynaecological anti-infective medications, medications used in anaemia, certain antibiotics, topical anaesthetics, analgesics, anxiolytics, antiparasitics, bronchodilators, vitamin D3 and infusion fluids. If it is necessary to use other drugs not listed in the regulations, that are also over the counter (OTC) category medicines, a nurse or a midwife can recommend them by making an appropriate note in the medical record. They may also issue prescriptions for medications that are not included in the regulations of the Minister of Health, in cases of repeat prescribing, that is, where a doctor has already issued an appropriate instruction for the nurse or the midwife. The only extant restriction to this entitlement concerns a specific group of very potent medications, narcotics and psychotropics. There was also a further restriction (during 2017 and 2018), that nurses and midwives were not permitted to issue prescriptions for medical devices, and this caused real impediments to nurses’ care for patients. For instance, cases such as the following required a doctor’s prescription: ongoing provision of wound treatment where subsidized categories of dressings were required; or, ensuring recurring access to blood glucose test strips in the treatment of diabetic patients. Yet in such cases a doctor may not have been present when the nurse was treating the patient, especially for instance, during in-home care. That restriction in the law was subsequently changed, and since 2019 nurses are permitted to prescribe medical devices.

Nurses and midwives in Poland may prescribe a medication and issue a prescription following a personal physical examination of the patient. Prescriptions may also be issued without a personal physical examination of the patient only if justified by evidence of the patient’s health reflected in the medical record. They therefore have the right to inspect the patient’s medical record and to obtain complete information from the doctor on the patient’s health, diagnosis, suggested diagnostic, therapeutic, rehabilitation and preventive methods and the foreseeable consequences of the measures taken, as far as is necessary for the nurses and midwives to determine the health services that ought to be provided to the patient. In other words, they may fulfil their entitlement to order and issue prescriptions without any formal obstacles. Additionally, nurses and midwives are entitled to obtain and process the data included in medical records.

Nurses and midwives are obliged to practice their profession with due diligence, in accordance with the rules pertaining to professional ethics, observing the patient’s rights, protecting the patient’s safety and making use of the indications of current medical knowledge. If a prescribing error is committed, civil liability may be considered; while in exceptional cases where there is a criminal offence, criminal liability may be considered. It is also possible for nurses and midwives to be held professionally responsible before a nurses’ and midwives’ court. Nurse prescribing and the validity of prescription issuance is overseen by the National Health Fund (NHF) which is also the government body which delivers funding for Poland’s free national health care services.

The study aimed to investigate attitudinal influences on, and actual results of, the early phases of nurse prescribing in Poland at the time it was introduced at the beginning of 2016. The paper analyzes the opinions of nurses, expected to be influential on nurses’ actual practices, prior to legislative change and compares this with actual nurse prescribing behaviours during the early years of implementing legal nurse prescribing. Analyzing the opinions of nurses prior to implementation was important, partly because there were no other studies available, and because whether a qualified nurse or midwife would practice as a prescriber was at their own discretion, not that of their employer. As the first study of its kind in Poland, the paper addressed a knowledge gap in the field, and provides baseline data analysis for subsequent research.

## 2. Materials and Methods

In order to understand the initial period of the implementation of nurse prescribing in the Polish health system the researchers conducted an analysis of the opinions of nurses and midwives while they were preparing themselves for prescribing, and then compared this data with an analysis of the actual patterns of prescribing by nurse prescribers. Data were obtained from two sources including nurse surveys and NHF files. In the first phase of the project, 819 responses, from a total of 1500 surveys distributed, were submitted by nurses and midwives who were participating in nurse prescribing training courses delivered across Poland, and were analyzed. In the second phase of the project all 191,620 nurse prescriptions refunded from the NHF budget during the first two years of the implementing period were analyzed.

### 2.1. Study of Nurses’ and Midwives’ Opinions

#### 2.1.1. Research Design and Study Setting

The first phase of the research entailed a national cross-sectional, targeted, voluntary questionnaire to seek nurses’ opinions during the period they were preparing themselves for prescribing. The research instrument was a 12 item validated questionnaire with 8 attitudinal questions (in five questions only one answer was possible, and in the remainder, more than one answer was possible) and four questions in a demographic section to record each respondent’s age, gender-identification, education level and level of seniority at work. Closed question format was used throughout. The Likert Scale was used in three questions. The questionnaire form also included an information section that explained the research project, and provided details on researcher and their institution, and included instructions on how to complete the questionnaire. The questionnaire design was intentionally brief (12 questions) to ensure as high a response rate as possible. The questionnaire was original and entirely purpose-designed for our research project and followed design guidelines in the international literature [9,10]. When designing the questionnaire, Poland’s specific cultural character was taken into consideration. We used a content validity measure: during the survey design, 6 scientists from Sociology Department were asked to rate the survey questions and categorize them as “essential”, “useful” or “not necessary”. For each question the Content Validity Ratio was calculated, and the results were from 0.6–0.9. The draft questionnaire was analyzed using a recognized Polish text comprehension tool (jasnopis.pl, SWPS University, Warsaw, Poland) to ensure that it was grammatically concise, and easy to understand; and the analysis result for all survey questions was “simple to understand”–gaining (7 points on a 7 point scale). The validation of the prepared questionnaire was started by measuring the time needed to complete it, and then questions about the clarity and intelligibility of its contents were asked. Doubts or suggestions on the part of the respondents were helpful in clarifying the questions contained in the questionnaire. The internal consistency of the questions using the Likert scale was examined using the Alpha-Cronbach coefficient; and a significant, high correlation was found between the results obtained for each question and the total number of points. The calculated ratio was high, at 0.85, which indicated there was a very high degree of internal consistency in the questionnaire. Then the tool was validated in a group of 10 nurses, all members of the Gdansk branch of the Polish Nursing Society, who had been invited to participate by e-mail. All their comments were taken into consideration. We conducted a pilot study to identify any potential problems or deficiencies in the research instruments prior to implementation with the full study group.

All the obtained results were subjected to statistical analysis using the STATISTICA 10.0 program (StatSoft. Inc., Palo Alto, CA, USA; www.statsoft.com) and Excel spreadsheets (Microsoft Corp., Redmond, WA, USA). The entire statistical analysis was carried out at the confidence level α = 0.05. The probability of making the first type of error consisting in rejecting the null (true) hypothesis was less than 0.05, which was considered as statistically significant.

#### 2.1.2. Study Population and Data Collection Process

When the composition of the questionnaire was finalized, paper format copies were completed by nurses and midwifes who were preparing to practise as either nurse independent prescribers or as nurse supplementary prescribers. The researchers calculated the required sample size for the questionnaire on the following bases. At the time of the project, there were 221,172 nurses and 25,938 midwives employed in Poland, making a total of 247,110 [11]. It was determined that a minimum sample size of 384 completed questionnaires were needed to achieve a confidence level of 95% and that the real value is within ± 5% of the surveyed value. The total participant numbers of 819 nurses and midwives exceeded this minimum required. In total, 1500 questionnaire forms were distributed, with a return rate of 54.6%. The research was conducted from July to December 2015, in the period following the announcement of the new law but before the law took effect. When considering the likely response rates for our survey, our method was informed by the findings of Sax, Gilmartin, and Bryant (2003) [12] that shows that when a survey has applicability to the lives of the respondents, they are more likely to respond. We targeted nurses and midwives who were already participating in the special Diploma courses on Nurse Prescribing to help nurses and midwives prepare for prescribing. We only selected those nurses and midwives who elected to participate in our targeted national survey (meaning the survey was exclusively aimed at nurses and midwives who were enrolled in the same official nurse prescribing course delivered at different accredited Universities across the country in 2015). The participants who were invited to take part in the study were drawn from the nurse prescribing courses organized in 2015 by five medical universities (Bydgoszcz, Gdansk, Kielce, Warszawa and Wroclaw) during the theory components of their courses. Contact data of the organizers of these courses was available on the official website of the Ministry of Health [13]. These diploma courses were funded mainly by the European Union, (Operational Programme Knowledge Education Development, Priority axis V Support for healthcare) commissioned by the Polish Minister of Health, and supported by the Polish Nursing Society, and offered nurses and midwives training in the prescribing of medications. Further, the inclusion criteria for participant selection were that the nurses or midwives were in active service, regardless of their level of education, and preparing for their role as prescriber. The participant’s level of education was not a criterion since nurses and midwives may complete an additional course in prescribing medicines and issuing prescriptions with a variety of prior higher education qualifications. Therefore, there were no exclusion criteria. The participants in our study were guaranteed full anonymity.

Attitudinal data were collected anonymously using self-administered paper-based surveys handed out to the nurse and midwife respondents. The questionnaires were distributed and collected anonymously by one of the researchers (a PhD student). The survey method constituted a targeted survey. The researchers received permission from the nurses and midwives’ course lecturers and from the Polish Nursing Society to distribute and collect the completed questionnaires during the theory components of their Nurse Prescribing courses. A closed and locked collection box was used for the completed questionnaires.

#### 2.1.3. Data Processing and Analysis

Questions with no answers (nonresponse item) were excluded from the analysis. Data collected was digitized manually and a unique number was generated for each questionnaire, by a person responsible for entering the data into an excel database. Coding and data entry accuracy was checked by a second person from the research team. The survey-generated data was analyzed by the nonparametric ANOVA Kruskal-Wallis test with the post-hoc of multiple comparisons of medium ranks. The interrelationships between the categorical variables were determined using chi-squared tests.

### 2.2. Prescribing Data Analysis: Retrospective Research

#### 2.2.1. Research Design and Study Setting

The second stage of the study was based on a secondary analysis of the data on all the reimbursed nurse prescriptions during the implementation period (2016 and 2017) from the public records held by the NHF. Prescription data was collected from the NHF. This process involved writing to the NHF with our research request, in which we were required to demonstrate that there was a significant public interest in the research findings that would result, before we were granted access to the data. We were also required to explain that ours was a scientific study which would generate neutral (objective) results showing trends and how implementation of nurse prescribing was working. In Poland, the National Health Fund does not make any analysis of its own data in this field, at least because they consider it too much work to analyze data contained in paper-based prescriptions. Once we had received both NHF permission, we were sent all the prescriptions on an encrypted CD (compact disc). Because of the nature of the data contained in the file and the fact that they included prescriptions from all around the country the researchers were required to ensure that the data was kept secure. The data CD received from the NHF was encrypted and the access code was provided to only one person from the research team. When not in use, the data CD was stored in a key-locked cabinet in a secure room with access limited to authorized personnel only. The prescriptions data we collected included information on the number and type of prescriptions issued across Poland, in an Excel file; and this data included the European Article Number (EAN) of each prescribed product. The data also included the number of the province from which the prescriptions originated. The first step was to decode the EAN number and assigned it to a specific medication. Using the statistical computing language R (R Core Team, R: A language and Environment for Statistical Computing, (R Foundation for Statistical Computing, Vienna, Austria)), scripts were created to map the information from the public data of the Office for Registration of Medicinal Products, Medical Devices and Biocidal Products onto the database developed for our project. Every year the President of the Office publishes a list of products authorized for sale within Poland. The lists contain the EAN codes for each medicinal product and identify the active substance for each EAN. Thus, we were able to identify the active substances for each prescription, and this data formed the basis of further analysis. Substances were assigned to groups from the ATC (anatomical-therapeutic-chemical) classification system, using Microsoft Excel. This was an extremely laborious phase of the raw data analysis that had to be performed manually because no computer programme exists that could identify the medicines and classify them into appropriate groups in the way we wanted. The overall data included information on each patient’s age and prescriber type, which required decoding. In prescription NHF files, each patient is characterized by a unique anonymizing code beginning with his/her year of birth. The embedded age data were used to determine the ages of patients in the study. The type of prescription (whether issued independently or on a physician’s order) was identified by the penultimate numeral of the prescription code number (as each prescription has its own individual and unique number). The penultimate digit in each prescription code is either a 5, meaning the prescription was issued by a nurse, or a 6 meaning it was a repeat prescription. However, the same prescription coding by the pharmacy does not indicate whether the prescription was related to either secondary or primary care, and the research data we received already aggregates the data from primary and secondary settings.

In the second phase of the project, we analyzed all 191,620 nurse prescriptions that were issued during the first two years of the implementation period (that were NHF-refunded prescriptions). Prescriptions had been generated in every province of Poland. Data on each prescription is initially generated by the pharmacies, sending a code number with each prescription that permits us to identify whether it was a supplementary or an independent prescription. However, the same prescription coding by the pharmacy does not indicate whether the prescription was related to either secondary or primary care. The survey-generated data was analyzed by the ANOVA Kruskal-Wallis nonparametric test. The interrelationships between the categorical variables were determined using chi-squared tests. The assumed significance level was α = 0.05.

#### 2.2.2. Ethical Considerations

The research project received Independent Bioethics Committee for Scientific Research at the Medical University of Gdańsk (ethical approval number 394/2015), including an assessment that the project constituted “non-invasive research”. All participants in the attitudinal phase of the project (the questionnaire) gave their informed and voluntary consent prior to participation. All data in the second phase of the project (prescribing data analysis) was anonymized and no individuals could be identified.

The study was conducted in accordance with the requirements of Poland’s data protection legislation. Participants received both written and oral information about the study. All the questionnaires were collected in locked boxes and subsequently stored in a locked office. Nurse prescribing data was stored in a strongbox in a locked office, and on a CD with a secure login password.

## 3. Results

### 3.1. Study of Nurses’ and Midwives’ Opinions

There were 804 women and 15 men involved in the questionnaire study (the first phase of the research), which reflects the actual gender ratio of working nurses in Poland [14]. 152 (18.7%) respondents were 22–29 years old (normally, a nurse with a bachelor’s degree starts work when they are 22); 95 (11.7%): 30–39 years old; 318 (39.2%): 40–49 years old; 228 (28.1%): 50–59 years old; 19 (2.3%) respondents were over 60 years old, and 7 respondents did not state their age.

Most respondents held a bachelor’s degree: 357 (43.7%); while 263 (32.2%) had a master’s degree; and the remainder (199, 24.1%) were graduates of medical secondary schools, qualifying under Poland’s former nursing education system. Hence all of the respondents would have been permitted to take a special course preparing them to prescribe medications or issue prescriptions. The group of nurses and midwives whose highest qualification was from medical secondary school constituted 24.1% of the respondents, but they also included persons who had a specialisation which permitted them to be enrolled on the course. The other respondents from the secondary school sub-group also had the possibility of completing the specialisation, which qualifies nurses to be prescribers, and therefore they were also eligible to participate in our study. The results are shown in Table 1.

The questionnaire first asked the respondents about the roles they play in patient pharmacotherapy processes which are understood as the necessary scope of practice for nurses who are prescribing or assisting with prescribing (it was possible to give multiple concurrent answers). The study subjects identified that their role was primarily in monitoring doctor’s indications (68%), educating patients about the administration of their medications (67.3%), and monitoring and reporting adverse reactions (65%). A smaller number of respondents identified the role of assisting doctors (performing supplementary activities) with the issuing of prescriptions (28.6%). Nurses also less frequently identified their role as conducting drug reviews (32.9%). The results are shown in Table 2.

While analyzing possible relationships between the practice role and the years of experience of the respondents, we found that there was a statistically significant difference between respondents with 31–40 years’ experience, who were more likely, and those with 0–10 years’ experience, who were less likely, to assist with the issuing of prescriptions, *H* test = 11.45; *p* < 0.05. However, those with the lowest level of experience (0–10 years) were found to monitor compliance with the doctor’s instructions significantly more often than other groups, *H* test = 13.79; *p* < 0.05.

Secondly, the questionnaire sought nurses’ and midwives’ opinions on whether they should issue prescriptions as part of their professional duties. Positive opinions to the new entitlements were demonstrated by 371 (45.3%) people answering: “strongly agree” and “agree”. 329 (40.2%) respondents expressed negative opinions related to them not being paid by either the NHF or their employers for undertaking new responsibilities. The remainder of the group did not express either positive or negative opinions. The distribution of answers is summarized in Table 3.

Our analysis revealed that age was not a factor in the respondents’ opinions on prescribing (*H* test = 5.43; *p* = 0.246); but we did find a correlation between the respondents’ years of experience and their opinion on prescribing (*H* test = 17.65; *p* < 0.05). To identify which groups showed significant differences from other groups, the Bonferroni method of multiple comparisons was used. The analysis showed that respondents with 11–20 years’ experience disagreed with prescribing by nurses and midwives significantly more often than respondents from all other groups (0–10 years, 21–30 years, 31–40 years and greater than 40 years). A correlation was also found between the opinions and the respondents’ levels of education. Respondents with a master’s degree expressed more favourable opinions on prescribing by nurses and midwives than those respondents who graduated from a medical secondary school, a medical college or from bachelor’s degree studies.

The third survey question concerned the respondents’ subjective assessment of whether their own preparedness for prescribing before entering the course was sufficient. According to 50% of the respondents, their knowledge and skills were unsatisfactory for the task ahead (with answers of “strongly disagree”: 156 people; and “disagree”: 254 people). 183 (22.3%) respondents “agreed” that their preparedness for prescribing was sufficient, while only 42 (5.1%) “strongly agreed” that their preparedness was sufficient. Nearly 23% (185 respondents) answered “neither agree nor disagree”.

Respondents identified two benefits for patients of nurse prescribing: in the treatment of chronic diseases (590 answers, 72.1%); and the management and treatment of acute pain and infections (229 answers, 27.9%).

Respondents were also asked for their opinions on what benefits they will draw from prescribing, and what dangers they foresee in the new entitlement (multiple answers could be given). The benefits they identified included:

a) Greater professional autonomy: 527 answers (64.3%);
b) Higher professional status: 348 answers (42.5%);
c) Higher remuneration: 219 answers (26.7%);
d) Greater work satisfaction: 186 answers (22.7%).

The dangers they identified included:

a) Greater legal responsibility and liability: 631 answers (77.3%);
b) Reduced time for patient care: 585 answers (71.4%);
c) No additional remuneration: 584 answers (70.9%);
d) Increased workload: 509 answers (62.1%);
e) Inadequate preparation: 385 answers (47%);
f) Conflicts in the therapeutic team: 240 answers (29.3%);
g) Lack of substantive support: 227 answers (27.7%).

Respondents were also asked which groups of medications they would be ready to prescribe independently, from within the choice of medications permitted by the legal regulations for nurse prescribing. Respondents indicated that they would independently issue prescriptions for analgesics in the first place. The results of the analysis are shown in Table 4.

The analysis demonstrated that there was a correlation between the educational level of respondents and their preference for prescribing medicinal products. Those with a master’s degree said they would issue a prescription for medications statistically significantly more often than respondents who graduated from medical secondary school, medical college or bachelor’s degree studies.

### 3.2. Prescribing Data Analysis: Retrospective Research

A secondary analysis, comparing the first year of implementation data obtained from NHF with that for the second year of implementation showed that the number of nurse prescriptions had been increasing. In 2016, there were 536 nurse independent prescriptions issued, and in 2017 there were twice as many, i.e., 1323. The number of nurse supplementary prescriptions issued in 2016 was 28,674, and in 2017 it was almost six times higher at 161,087. Prescribing rates differ from one province to another because of population differences and the varied numbers of prescribers in each province. These results were further broken down by province. It is evident that in Poland, nurse supplementary prescribing, on doctors’ orders, was more frequent than nurse independent prescribing, in the NHF prescribing data during the first two years after the introduction of the new law.

Nurse independent prescribing may apply to specifically defined medications and medical devices (dressings and blood glucose test strips). During the implementation period nurse independent prescriptions were mainly issued for dressings. 321 prescriptions were issued for reimbursed dressings in 2016, and in 2017 there were 665 such prescriptions. In the medication group, prescriptions for oral antibiotics predominated. Table 5 below summarizes the results. During the questionnaire phase of the research, nurses were asked which drugs they anticipated prescribing and they thought they would be mainly prescribing anti-emetics, and only rarely antibiotics. However, the actual case was exactly the opposite, as the prescriptions data showed that nurses issued relatively few prescriptions for anti-emetics, and instead, mainly prescribed antibiotics.

**Figure 1:**
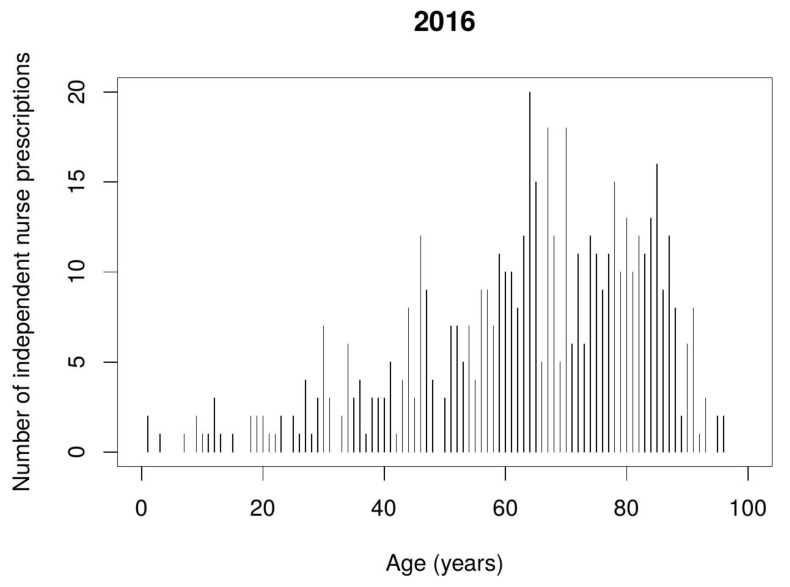
Number of nurse independent prescriptions depending on the patient’s age, during the first year of implementation.

**Figure 2:**
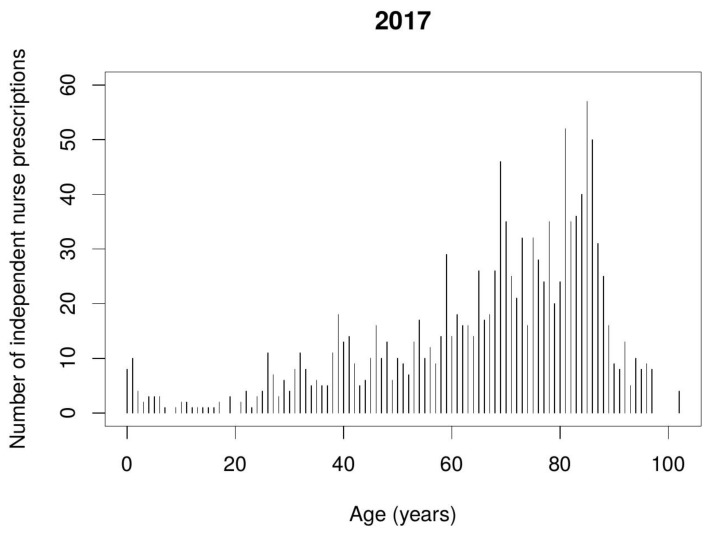
Number of nurse independent prescriptions depending on the patient’s age during the second year of implementation.

Data from the Polish NHF indicates that nurse independent prescribing mostly takes place in relation to pain management and combating infections. Prescriptions were issued to patients of diverse ages, but mostly to the elderly, i.e., 65–85 year-olds. Figure 1 and Figure 2 show the number of nurse independent prescriptions depending on the patient’s age.

In nurse supplementary prescribing, the predominant medications were those used in the treatment of chronic conditions, i.e., for hypertension to lower cholesterol levels, and for cardiovascular disorders. There were also numbers of medications used for digestive tract disorders. The fewest prescriptions were issued for proctological, gynaecological (by midwives) and antiparasitic medications. All results are shown below in Table 6.

In the category ‘medical devices’ the following were prescribed: in 2016; 403 prescriptions for dressings, 862 prescriptions for blood glucose test strips; and in 2017: 1429 prescriptions for dressings and 4578 prescriptions for blood glucose test strips. In addition, prescriptions for foods for special dietary purposes were issued (in 2016: 51 prescriptions; in 2017: 131 prescriptions).

**Figure 3:**
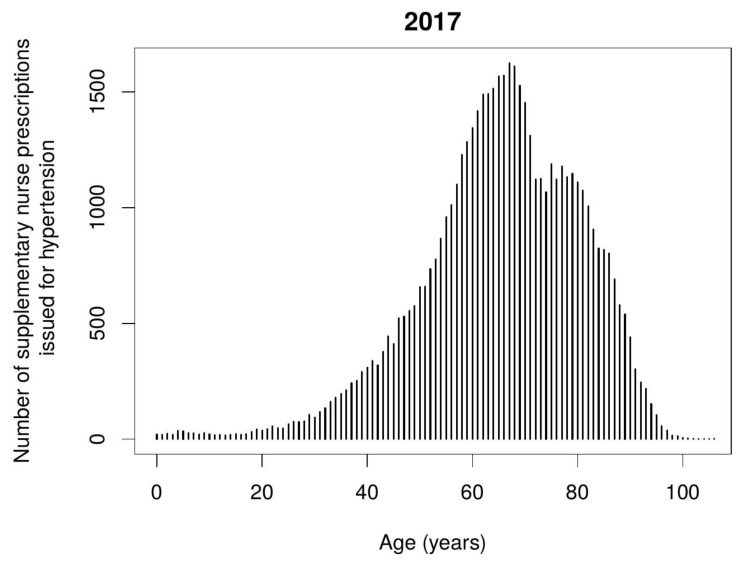
Number of nurse supplementary prescriptions issued for hypertension therapy during the second year of implementing period depending on the patient’s age.

The most frequently issued nurse supplementary prescriptions were for medications used for hypertension. The diagram (Figure 3) below shows which age groups these prescriptions were issued to, with the greatest frequently during 2017. Prescribing for the elderly in the 60–80 age group predominates.

**Figure 4:**
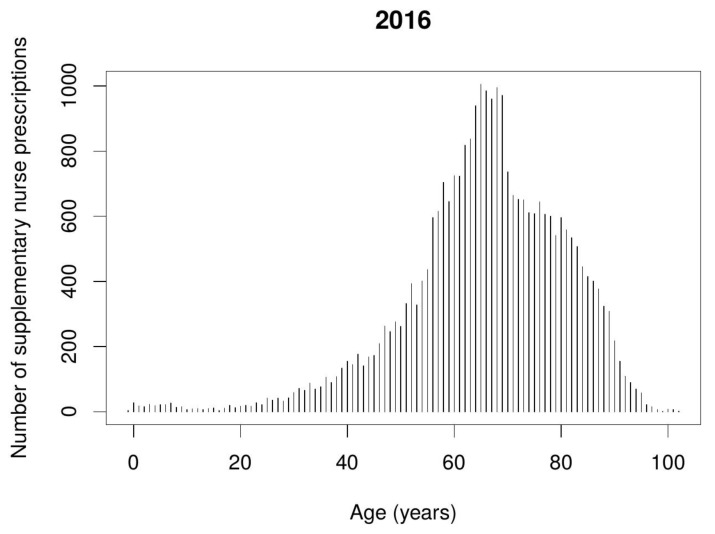
Number of nurse supplementary prescriptions depending on the patient’s age during the first year of implementing period.

**Figure 5:**
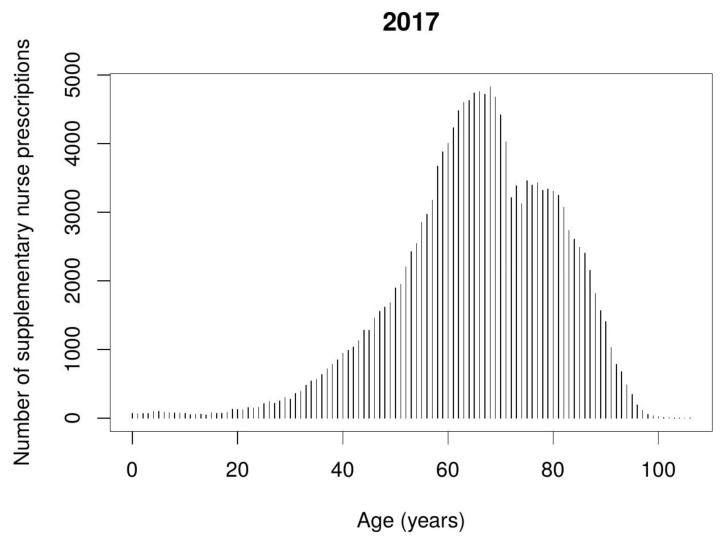
Number of nurse supplementary prescriptions depending on the patient’s age during 2017.

Nurse supplementary prescribing was predominantly applied in the treatment of elderly patients aged 60–80 years old. Figure 4 and Figure 5 show the number of nurse supplementary prescriptions according to the patient’s age.

## 4. Discussion

Nurse prescribing already has a long history in many countries [15]. Most studies emphasize nurse prescribing has a positive influence on health care results [16,17,18,19]. Some studies show that when comparing the clinical outcomes in patients arising from doctors’ prescribing with those arising from nurses prescribing, there are no significant differences [1].

To date, there have not been any other analyses of nurse prescribing in Poland during the implementing period. Some Polish research has reported the positive opinions of nurses about prescribing [20,21,22], but that research did not assess nurse prescribing practice. A better understanding of the determinants of prescribing patterns in Poland may help shape future workforce policy in the medical sector and contribute to the design and implementation of optimal and cost-effective initiatives to improve care quality.

The picture emerging from our own research indicates that even though they were enrolled in a specialist nurse prescribing course, which was a pre-requisite for authority to prescribe, during this preparation period nurses were not convinced that they should issue prescriptions (54.7% had either no opinion or negative opinions of the new entitlement). Additionally, nurses identified their lack of proper preparation as a barrier to the assimilation of nurse prescribing (47%). It was possible that some of the course participants, prior to completion, might have felt the course was more of a licensing formality than a substantive learning opportunity. However, even those with negative opinions participated in the free Diploma training courses. This might suggest they were unaware that though entitled, their own practice of nurse prescribing was optional rather than mandatory. It might also indicate their positive disposition towards their own professional development. Our study shows other links between professional development and prescribing in that nurse prescribing was more likely to be associated with nurses’ higher professional status (42.5%) and greater professional autonomy (64.3%). Though many respondents rated their preparedness for the new role as poor (23% offered no opinion on their knowledge and skills, and more than 50% were of the opinion that their knowledge and skills were poor), in the first two years of nurse prescribing the number of prescriptions issued grew more than five-fold. It is worth noting that nurse prescribing is generally associated with master’s degree holders [23].

The experience of many countries, such as the USA, Canada, Australia, Ireland, New Zealand, Sweden, and the UK, where similar entitlements have been introduced, shows that they have brought positive effects for patients, including appreciating the quality of the information they receive about additional therapeutic procedures and gaining high levels of satisfaction from the treatment used [24,25]. Polish nurses have expressed concern about possible time reductions in patient care (71.4%); and they believed their workload would increase (62.1%). These opinions might suggest that they are overburdened with current duties and have no time for additional therapeutic activities.

Compliance with therapeutic indications is an important element of pharmacotherapy. International research has shown that the participation of nurses in prescribing improves compliance [26]. Nurse prescribing is most beneficial in the treatment of the elderly (60–85 years old); nurse prescribers also help to reduce polypharmacy in the elderly; and our research also found that nurse supplementary prescribing was predominantly applied in the treatment of elderly patients aged 60–80 years [27].

Research indicates that in countries with nurse prescribing, it mitigates situations of medical staff shortages; enables interdisciplinary cooperation; and bridges the distance between nurses and other professions, thus benefiting the whole team and allowing doctors to focus on more complicated clinical cases [28,29]. Concurrently, the practice reduces the professional distances between health care providers [30]. In our own research, the respondents who had no experience of prescribing at the time of our study pointed out that conflicts might arise in the therapeutic team (29.3%); and some other studies also point to the possibility of conflicts [31]. Polish nurses have also expressed concerns about being burdened with additional work (62.1% respondents), which would contribute to negative outcomes for the whole therapeutic team.

In Poland, respondents identified the increased risk of legal liability, because of the professional fault principle in Polish law, as a negative consequence of nurse prescribing (77.3%). In addition, respondents believed that the new roles are not associated with additional remuneration (70.9%). It is not mandatory under Polish law for an employer to raise the salary of a nurse prescriber in the public system. In the private sector, the employer’s pay system choices are unconstrained. In contrast, respondents did expect that their new prescribing duties would enhance their professional status (42.5%) and work satisfaction (22.7%). The new entitlement is linked to increasing professional autonomy (64.3%). These benefits have also been reported in other studies, including those by Carey et al. and Scrafton et al. [32,33].

Poland has recently introduced the role of doctor’s assistant. Previously this role was fulfilled by nurses; and as for the time being there are few medical assistants, nurse prescribing can serve as a legal equivalent, in that nurses may issue a prescription and the physician only signs it. Evidence from our own studies shows that this assisting role in the issuing of medical prescriptions was practised by 28.6% of respondents.

The Polish practice of nurse prescribing is mostly for nurse supplementary prescriptions issued on a doctor’s orders, and mostly for the elderly in the treatment of chronic diseases, especially hypertension. Nurse prescribers in the UK, New Zealand and the Netherlands are playing a significant therapeutic role for senior citizens with chronic diseases [27,34,35]. In Ireland, the most frequently prescribed medications are analgesics and anti-inflammatory medications (containing paracetamol and ibuprofen), vaccines and antibiotics for adults [36].

The potential limitations of this work should be noted. In the questionnaire phase of the study the precondition for inclusion in the study was that participants had to have attended a nurse prescribing training course. However, we did not assess whether all participants had successfully completed said course and become certified prescribers. Another limitation is that in Poland there is no general record of all nursing prescriptions, only those funded by the NHF; and prescriptions that were paid for in full by patients were excluded from our study. The opinions survey was carried out very shortly after the announcement that nurses would have new prescribing powers in Poland, and therefore, further studies would be needed to determine whether opinions have changed over time and to understand those changes.

## 5. Conclusions

Nurse prescribing is a recent development in Poland. Nurses have had misgivings about their new role, assessing their preparedness as poor, and yet over the initial two years of legalized nurse prescribing the number of nurse prescriptions increased five-fold. In the period of vacatio legis, (i.e., the time given for nurses to prepare for their new role), nurses identified potential problems arising because of a reduction in the time available for caring for patients and an excessive increase in nurses’ own workload. Our study found that nurse independent prescribing occurs mostly in the treatment of pain and infections of elderly patients. The research demonstrated that nurses issued prescriptions mostly on doctor’s orders, and in the treatment of elderly patients’ chronic disorders. Further research is needed to determine whether nurse prescribing can reduce polypharmacy in the elderly. Other implications based on our findings and the limitations of the study include the need to evaluate the nurse prescribing course outcomes to identify if participants felt at the end of the course whether they were prepared to be a prescriber; knowing through further research about the effects of their prescribing, such as benefits to nurses and to patients (by investigating prescribers rather than non-prescribers); and evaluating of the consequences of nurses and midwives being able to choose whether to prescribe, to understand how many are electing to prescribe.

## Figures and Tables

**Table 1:** Survey participants’ demographic data.

Respondents Characteristics	*N*	*%*
Gender		
Female	804	98.2
Male	15	1.8
Age		
20–29 years	152	18.7
30–39 years	95	11.7
40–49 years	318	39.2
50–59 years	228	28.1
More than 60 years	19	2.3
Education		
Medical secondary school	199	24.1
Bachelor	357	43.7
Master’s degree	263	32.2
Years of practice		
0–10 years	192	23.6
11–20 years	141	17.3
21–30 years	279	34.3
31–40 years	187	23
More than 40 years	20	1.8

**Table 2:** Distribution of respondents’ answers to the question about the patient pharmacotherapy roles they fulfilled, *n* = 819.

Manner of Involvement in Patient Pharmacotherapy	*n*	%
Checking compliance with doctor’s instructions	557	68.0
Monitoring and reporting adverse drug reactions	484	65.0
Educating patients about administration of their medications	522	63.7
Conducting drug reviews	270	32.9
Assisting in prescribing	234	28.6

**Table 3:** Distribution of answers to the question “Should nurses issue prescriptions?” *n* = 819.

Should Nurses Issue Prescriptions?	*n*	%
Strongly agree	157	19.2
Agree	214	26.1
Neither agree nor disagree	119	14.5
Disagree	159	19.4
Strongly disagree	170	20.8

**Table 4:** Types of medicinal products that the respondents anticipated prescribing.

What Medicines Are You Likely to Prescribe	Strongly Agree		Agree		Neither Agree nor Disagree		Disagree		Strongly Disagree	
	*n*	%	*n*	%	*n*	%	*n*	%	*n*	%
Analgesics	309	37.8	307	37.5	48	5.8	49	5.9	106	13.0
Vitamins (applies to midwives)	286	35.0	222	27.1	92	11.2	56	6.8	163	19.9
Anti-emetics	234	28.6	295	36.0	97	11.8	75	9.2	118	14.4
Systemic anti-infective drugs (antibiotics)	206	25.2	272	33.2	112	13.6	82	10.1	147	17.9
Infusion fluids	166	20.2	251	30.6	98	12.0	129	15.8	175	21.4
Topical anaesthetics	137	16.6	286	34.9	103	12.6	110	13.5	183	22.4
Antiparasitics	123	14.9	251	30.7	140	17.1	133	16.3	172	21.0
Gynaecological drugs	115	13.9	143	17.5	171	20.9	152	18.6	238	29.1
Bronchodilators	106	13.0	199	24.3	140	17.2	168	20.5	204	25.0
Topical anti-infective drugs	91	11.0	215	26.3	143	17.5	168	20.5	202	24.7
Drugs for anaemia	69	8.4	160	19.6	150	18.3	213	26.0	227	27.7
Anxiolytics	38	4.7	115	14.0	262	32.0	171	20.91	233	28.4

**Table 5:** Groups of medicines prescribed by nurse independent prescribers during the implementation period.

Medicines Prescribed Independently Per Year	First Year	Second Year
	No. of Prescriptions	No. of Prescriptions
Anti-emetics	7	35
Topical anti-infective drugs	0	0
Gynaecological drugs	4	10
Drugs for anaemia	1	4
Systemic anti-infective drugs (antibiotics)	63	170
Topical anaesthetics	0	0
Analgesics	18	89
Anxiolytics	0	0
Antiparasitics	17	15
Bronchodilators	2	4
Vitamins (applies to midwives)	2	5
Infusion fluids	0	0

**Table 6:** Number of nurse supplementary prescriptions issued for specific groups of medicinal products during the implementation period.

Supplementary Prescribed Medicines Per Year	First Year	Second Year
	No. of Prescriptions	No. of Prescriptions
Used for hypertension	9632	53,675
Cholesterol-lowering	4109	22,602
Antidiabetic	2576	13,923
Used for cardiovascular disorders	2298	11,795
Used for digestive disorders	1915	10,989
Thyroid hormones	963	6042
Used for urogenital disorders	701	4371
Neurological	584	3607
Insulins	562	3827
Anti-infective	530	3336
Used for bone and bone and skeletal system disorders	510	2606
Inhalation aerosols and medicines used for respiratory disorders	481	3064
Anti-emetics	413	2592
Used for haematopoietic disorders	402	2317
Antiallergic	321	1942
Psychiatric	315	1976
Ophthalmic	310	1899
Vitamins and microelements	297	1335
Hormones other than sex hormones	129	785
Antibiotics	91	551
Dermatological	71	524
Sex hormones	47	500
Immunomodulatory medicines	44	336
Laryngological	41	255
Antiparasitic	8	52
Gynaecological	3	19
Proctological	2	6
Vaccines	1	1
